# Toxicity assessment of potassium bromate and the remedial role of grape seed extract

**DOI:** 10.1038/s41598-022-25084-7

**Published:** 2022-11-28

**Authors:** Emine Yalçin, Kültiğin Çavuşoğlu

**Affiliations:** Department of Biology, Faculty of Arts and Sciences, Giresun University, 28200 Giresun, Turkey

**Keywords:** Biochemistry, Genetics

## Abstract

In this study, the multiple toxic effects of potassium bromate were investigated in *Allium cepa* L., an indicator test material. In addition, the toxicity-reducing effects of grape seed extract (GSE) were tested. The toxicity was investigated by some physiological (germination percentage, root length, weight gain, relative injury rate), cytogenetic [mitotic index (MI), micronucleus (MN), and chromosomal abnormalities (CAs)], biochemical [malondialdehyde (MDA), superoxide dismutase (SOD), catalase (CAT), glutathione (GSH) levels] and anatomical parameters. *A. cepa* bulbs were divided into 6 groups as control and five treatment groups (Group II: 465 mg/L GSE, Group III: 930 mg/L GSE, Group IV: 100 mg/L potassium bromate, Group V: 100 mg/L potassium bromate + 465 mg/L GSE, Group VI: 100 mg /L potassium bromate + 930 mg/L GSE). The bulbs were germinated for 72 h and at the end of the period the bulbs were subjected to routine preparations and made ready for analysis and measurements. As a result, potassium bromate exposure caused statistically significant (p < 0.05) decreases in all physiological parameter values. Potassium bromate application decreased MI by 41.6%, and increased the MN and CAs frequencies. CAs such as fragment, sticky chromosome, and vagrant chromosome, unequal distribution of chromatin, reverse polarization, nuclear bud and disordered mitosis were induced in root meristem cells. The mechanism of potassium bromate genotoxicity has been associated with DNA-potassium bromate interaction supported by spectral shift. Potassium bromate caused a decrease in GSH levels and an increase in MDA, SOD and CAT levels, thereby disrupting the antioxidant/oxidant balance in root tip cells. GSE administration in two different doses together with potassium bromate reduced the toxic effects and caused improvements in all parameters examined. The most significant reduction in toxicity was in group VI, which received 930 mg/L GSE, and there was an improvement about 18% in MI levels and an improvement about 44% in GSH levels in this group. While GSE application increased physiological parameters and GSH levels, it decreased MDA, SOD, CAT levels, MN and CAs frequencies. As a result, it has been determined that potassium bromate causes multi-directional toxicity at high doses and *A. cepa* is a very reliable indicator in determining this toxicity. In addition, GSE extract has been found to have a strong role in reducing the toxicity induced by potassium bromate.

## Introduction

Potassium bromate (KBrO_3_) is a colorless, odorless, tasteless and white crystalline oxidizing chemical used as a thickener in many industrial processes. It is widely used in the food industry to adjust the elasticity of dough, especially for bread making. It is used to obtain less extensible but more elastic dough by oxidizing the sulfhydryl groups of the gluten protein in the flour. Potassium bromate keeps the carbon dioxide produced by the yeast, making the dough elastic. The general purpose of the use is to make the bread rise in the oven, to preserve the aroma, to improve the taste and appearance and to increase the volume and texture of the bread. In addition, adding potassium bromate to freshly ground flour also extends the shelf life of the flour. Studies have shown that potassium bromate degrades the basic vitamins (B vitamins, A2, thiamine, riboflavin and niacin), minerals (Fe, Se and Mg) and fatty acids in bread, reducing the nutritional quality of bread and various health problems occur in people who consume this bread. Therefore, the maximum potassium bromate concentration allowed in bread by the US Food and Drug Administration (FDA) is 0.02 μg/g (0.02 mg/kg). In some countries (Nigeria, Canada, Argentina, China, Korea and Sri Lanka), although the use of potassium bromate in bread is completely banned and other non-toxic, thickening alternatives such as ascorbate are recommended, bakers in many countries still use potassium bromate to enrich the breads. Potassium bromate is also used in the manufacture of fish paste, cheese, and the production of fermented beverages (in beer malt), the manufacture of hair lotions, laboratory reagents and explosives. Potassium bromate is very soluble in water (75 g/L at 25 $^\circ$C) and very stable in water solution at room temperature. At temperatures above 370 $^\circ$C, it decomposes with the release of oxygen and toxic fumes^1–4^. Potassium bromate is used not only as an oxidizing agent in the production of bread, but also as a neutralizer in the manufacture of textiles and cosmetics industry. It can also occur as a pollutant in drinking water due to the conversion of bromide, which is naturally found in water, to bromate by ozone, which is used as a water disinfectant. In other words, it is one of the most common disinfection by-products of surface water. In this way, it can contaminate the environment and the organisms that spread in this environment. The use of water containing potassium bromate for irrigation purposes in agricultural applications poses a risk to the plants in the ecosystem and the living things that feed on these plants^5,6^. With the consumption of agricultural plants grown on soils contaminated with potassium bromate, toxicity progresses along the food chain. Potassium bromate, an oxidizing agent, causes various toxic effects by creating oxidative stress in living things. Many studies in the literature report cytotoxicity, genotoxicity, mutagenicity, carcinogenicity and biochemical toxicity caused by potassium bromate. The acute effects of potassium bromate toxicity in humans are nausea, vomiting, diarrhea and abdominal pain. Its chronic effects are oliguria, anuria, deafness, vertigo, hypotension, central nervous system depression, hearing loss, renal failure and thrombocytopenia^7,8^. Studies in which the toxic effects on plants and the solution proposals applied to eliminate these effects are reported are not at the desired level yet. In this study, the toxic effects of potassium bromate on *A. cepa*, an indicator plant, and the toxicity-reducing effect of grape seed extract (GSE) application were investigated.

In recent years, plant extracts such as lycopene, green coffee, green tea, sage, nettle, *Ginkgo biloba* L., pomegranate, turmeric, ginger have been used in studies on reducing the toxicity caused by chemical agents. GSE, one of the toxicity-reducing extracts, is a natural extract obtained from the seed of *Vitis vinifera* L. GSE is a rich source of proanthocyanidins. It also contains oligomers (catechin and epicatechin), fatty acids (linoleic acid, oleic acid, linolenic acid and palmitoleic acid), tocols (fetopherols and tocotrienols), phenolic compounds (flavan-3-ol, phenolic acid, anthocyanin, flavonol and hydroxycinnamic acid), phytosterols (β-sitosterol, stigmasterol, campesterol, Δ-5-avenasterol and Δ-7-sitosterol) and minerals (Fe, Ca, Zn and Cu). Therefore, GSE is a powerful antioxidant and has a free radical scavenging effect. It protects the body against internal and external free oxygen radicals^9–12^. In the literature, the protective property of GSE against different toxicity has been reported in many studies and it has been reported to be protective against induced oxidative stress^13^, genotoxicity^14^ and cytotoxicity^15^.

There is no study in the literature reporting the effect of GSE, which has a protective role against the toxicity of various agents, against potassium bromate toxicity. In this study, the toxic effects of potassium bromate, which is contaminated to the environment from various sectors, on *A. cepa* and the toxicity-reducing effect of GSE were investigated. Biochemical, cytogenetic, anatomical and germination-related parameters were investigated to determine the toxicity and this toxicity-reducing effect. The interaction of potassium bromate and DNA was investigated by spectroscopic analysis and genotoxic effects were supported by the spectral shift results.

## Materials and methods

### Experimental materials and chemicals

**Figure 1:**
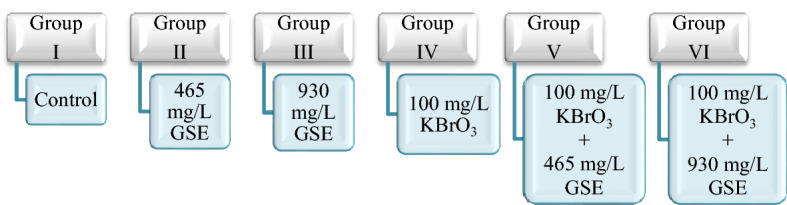
Experimental groups. GSE: grape seed extract, KBrO_3_: potassium bromate.

*A. cepa* bulbs (2n = 16), which is a bio-indicator organism, and GSE (*V. vinifera*—Sepe Natural—930 mg × 200 capsules) were used as test material. Commercially purchased GSE content consists entirely of grape seeds and does not contain any additives. Potassium bromate (KBrO_3_-Merck-CAS No: 7758-01-2) was used as a chemical toxic agent. *A. cepa* bulbs were divided into 6 groups and the groups are given in Fig. 1. Potassium bromate dose used in the experimental stages was chosen as 100 mg/L, which was reported to have a toxic effect in the previous studies^5^. GSE doses were chosen in the dose range where a protective effect was observed in *A.cepa* in the previous study^16^. Commercially available powdered GSE was extracted before the experimental steps. 10 g of GSE powder was incubated with 200 mL of dH_2_O for 12 h at room temperature. The residues obtained after the evaporation of the solvent at the end of the incubation were used as extract in all experimental stages. Experimental research on plant samples, including the supply of plant material, complies with institutional, national and international guidelines and legislation.

**Figure 2:**
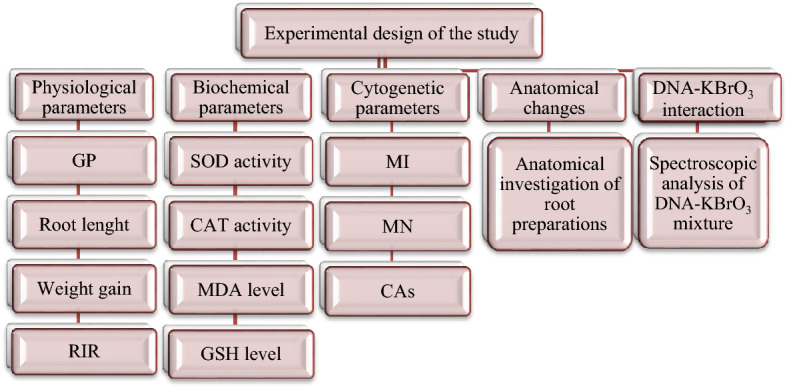
Experimental stages of study. GP: germination percentage, RIR: relative injury rate, SOD: superoxide dismutase, CAT: catalase, MDA: malondialdehyde, GSH: glutathione, MI: mitotic index, MN: micronucleus, CAs: chromosomal aberrations.

The bulbs were placed in sterile glass beakers and germinated continuously for 72 h at room temperature. The germination process was carried out by using tap water for the bulbs in the control group and using 100 mg/L potassium bromate and two different doses of GSE (465 mg/L and 930 mg/L) for the bulbs in the other five groups. During 72 h of germination, beakers were checked every 24 h and necessary solution additions were made. At the end of the period, the bulbs were washed in distilled water and prepared for analysis with the help of routine preparation techniques. At the end of the germination period, the bulbs were washed with distilled water and made ready for toxicity tests^17^. All parameters tested during the study are given in Fig. 2.

### Physiological parameters

The following criteria were taken as the basis for the measurement of physiological parameters.

- Root elongation was determined by measuring the length of the radicle, which is the embryonic root region and forms the root in the adult plant, with a millimetric ruler.
- Weight gain was determined by weighing the bulb weights before and after the treatment.
- Germination percentage (GP) was determined with the help of Eq. (1). ^18^
- Relative injury rate (RIR) was calculated by using Eq. (2).
  1 $${\text{GP }}\left( \% \right) \, = \, \left[ {{\text{Number of germinated seeds}}/{\text{Total number of seeds}}} \right] \, \times { 1}00$$
  2 $${\text{RIR }} = \, \left[ {{\text{GP}}\% {\text{ in control }}{-}{\text{ GP}}\% {\text{ in each group}}} \right]/\left[ {{\text{GP}}\% {\text{ in control}}} \right]$$

### Cytogenetic parameters

For MN and CAs analysis, the root tips were cut into 1 cm lengths, fixed in Clarke solution (3 volumes ethyl alcohol and 1 volume glacial acetic acid) for 2 h and washed in 96% ethanol for 15 min. Root tips were hydrolyzed in 1 N HCl in an oven at 60 °C for 17 min. At the end of the period, root tips were kept in 45% glacial acetic acid for 30 min. Then, root tips were stained with acetocarmine for 24 h. One of the dyed root tips was placed on a slide, pressed lightly with the help of a coverslip, crushed and examined under the Irmeco IM-450 TI model research microscope^19^. MN and CAs frequencies were determined by two different observers and abnormalities were photographed at × 500 magnification. Detection of MN in root tip meristem cells was performed according to three criteria proposed by Fenech et al.^20^. MI, which shows the mitotic activity, was calculated with the help of Eq. (3).

3 $${\text{MI }}\left( \% \right) \, = \, \left[ {{\text{Number of cells undergoing mitosis}}/{\text{Total number of cells}}} \right] \, \times { 1}00$$

### DNA-KBrO_3_ interactions

To elucidate the cytogenetic effects of KBrO_3_, the UV spectrum of the DNA and DNA-KBrO_3_ complex were investigated. For this purpose, DNA was isolated from *A. cepa* root tip cells and then used for spectral measurements. DNA isolation from *Allium* root samples was carried out according to the method suggested by Çavuşoğlu and Yalçın^21^. DNA-KBrO_3_ interaction was evaluated by investigating the change in absorbance of DNA and DNA- KBrO_3_ mixtures (1:1, 1:2). The UV absorption spectrum was obtained in the range of 240–280 nm. UV absorption spectra were recorded on the Mapada UV-6100PCS double-beam spectrophotometers.

### Biochemical analyzes

#### MDA and GSH levels

MDA level of each group was determined according to the method suggested by Unyayar et al.^22^. 0.5 g root tip were homogenized in 1 mL of 5% trichloroacetic acid solution. The homogenate was transferred to a new test tube and centrifuged at 12.000 g for 10 min. Equal volumes of supernatant and 0.5% thiobarbituric acid were transferred to a new test tube and incubated in 20% trichloroacetic acid solution at 96 °C for 30 min. Afterward the test tube was placed in an ice bath and centrifuged at 10.000 g for 5 min. The absorbance of the supernatant was measured at 532 nm and the MDA level was expressed as μM/g FW. GSH levels in homogenates were measured by determining the acid-soluble sulfhydryl level according to the method proposed by Vecchia et al.^23^ and expressed as µMol/g.

#### SOD and CAT activities

Before SOD and CAT activity measurements, enzyme extraction from root tips was carried out at + 4 °C. 0.5 g root tip was washed with distilled water and homogenized in 5 mL of monosodium phosphate buffer (50 mM, pH 7.8), centrifuged at 10.500 g for 20 min, and the supernatant was used for activity measurements^24^. SOD activity was measured according to the method proposed by Beauchamp and Fridovich^25^. The reaction solution with a total volume of 3 mL was prepared (1.5 mL 0.05 M monosodium phosphate buffer, 0.3 mL 750 µM nitroblue tetrazolium chloride, 0.3 mL 130 mM methionine, 0.3 mL 20 µM riboflavin, 0.3 mL 0.1 mM EDTA-Na_2_, 0.28 mL deionized water, 0.01 mL enzyme extract, 0.01 mL %4 insoluble polyvinylpyrrolidone). The reaction was started by placing the tubes under two 15 W fluorescent lamps for 10 min and ended by keeping the tubes in a dark environment for 10 min. Absorbance was measured at 560 nm and SOD activity was expressed as U/mg FW^24^. CAT activity measurements were carried out according to the method proposed by Beers and Sizer^26^. CAT activities were measured in UV–VIS spectrophotometer at room temperature by preparing a reaction solution (0.3 mL 0.1 M H_2_O_2_, 1.5 mL 200 mM monosodium phosphate buffer and 1.0 mL distilled H_2_O) with a total volume of 2.8 mL. The reaction was initiated by the addition of 0.2 mL of enzyme extract. CAT activity was measured by following the decrease in absorbance at 240 nm as a result of hydrogen peroxide (H_2_O_2_) consumption and expressed as OD_240nm_ min/g^24^.

#### Detection of anatomical damages

Root tips were washed with distilled water and cut into 1 cm lengths. The cut root tips were placed between a soft foam material and cross-sections were taken with the help of a sterile razor blade in one stroke. The sections taken were placed on a slide, stained with 5% methylene blue for 2 min, covered with a coverslip, examined under the Irmeco IM-450 TI model research microscope and photographed at × 200 magnification^27^.

#### Recovery effects of GSE

The recovery effects (RE) of GSE used at two different doses were calculated separately. In the determination of the RE, the data of Groups V and VI; the data of the 100 mg/L KBrO3-treated group and the data of the control group were taken as the basis. RE of GSE was calculated using Eq. (4).

4 $${\text{Recovery effect }}\% \, = \, \left[ {\left( {{\text{D}}_{{1}} - {\text{D}}_{{2}} } \right) \, /\left( {{\text{D}}_{{3}} - {\text{D}}_{{2}} } \right)} \right] \, \times { 1}00$$

(D_1_: data of Group V or VI, D_2_: data of Group IV, D_3_: data of control group).

### Statistical analysis

Statistical analyzes were performed with the help of SPSS Statistics 22 (IBM SPSS, Turkey) package program. Obtained values are shown as mean ± standard deviation (SD). Statistical significance between the means was determined using the One-way Anova and Duncan tests and was considered statistically significant when the p value was less than 0.05.

## Results and discussion

### Alterations in physiological parameters

The effects of potassium bromate and GSE applications on selected physiological parameters are shown in Table 1. The highest germination, root elongation and weight gain values were determined in the control group (Group I) and Group II and Group III, which were treated with two different doses of GSE. There was no statistically significant difference between the physiological parameter values observed in these groups (p > 0.05). Potassium bromate exposure at a dose of 100 mg/L caused statistically significant (p < 0.05) decreases in the physiological parameter values in Group IV. Compared to the control group (Group I), this reduction was 53% for germination, 3.48 times for root length and 5.90 times for weight gain. In Group V and Group VI, where potassium bromate and GSE were applied together, a statistically significant (p < 0.05) increase was observed again in the selected physiological parameter values. These improvements were directly proportional to the applied GSE doses and higher in Group VI where 930 mg/L GSE was applied. Compared to Group IV, germination increased by 23%, root length by 2.20 and weight gain by 4.10 times in Group VI. Despite these increases, the values did not reach the control group levels. When the relative injury rates calculated by using germination percentages were examined, it was observed that the highest damage occurred in Group IV-treated with 100 mg/L KBrO_3_. With the application of 930 mg/L GSE, the injury rate decreased and regressed to 0.3.

**Table 1:** The effects of KBrO_3_ and GSE on physiological parameters.

Groups	GP (%)	Root length (cm)	Weight gain (g)	Initial weight (g)	Final weight (g)	
Group I	100	8.70 ± 1.84^a^	+ 6.50^a^	7.56 ± 1.24	14.06 ± 1.82	
Group II	96	8.50 ± 1.82^a^	+ 6.74^a^	7.50 ± 1.26	14.24 ± 1.85	
Group III	98	9.10 ± 1.86^a^	+ 6.92^a^	7.44 ± 1.22	14.36 ± 1.88	
Group IV	47	2.50 ± 1.10^d^	+ 1.10^d^	7.63 ± 1.30	8.73 ± 1.34	
Group V	56	3.80 ± 1.35^c^	+ 2.45^c^	7.65 ± 1.32	10.10 ± 1.53	
Group VI	70	5.50 ± 1.58^b^	+ 4.51^b^	7.45 ± 1.20	11.96 ± 1.58	

Group I: Control, Group II: 465 mg/L GSE, Group III: 930 mg/L GSE, Group IV: 100 mg/L KBrO_3_, Group V: 100 mg/L KBrO_3_ + 465 mg/L GSE, Group VI: 100 mg/L KBrO_3_ + 930 mg/L GSE. Values are shown as mean ± SD. 50 bulbs were used for germination percentage and 10 bulbs were used for root length and weight gain. The averages shown with different letters ^(a-d)^ in the same column are significant at p < 0.05. GP: germination percentage, RIR: relative injury rate.

In the literature, the number of studies investigating the physiological toxicity of potassium bromate and its derivatives in different plant species is quite limited. However, the results obtained in these studies show parallelism with our findings. For example, Sahin et al.^28^ reported that potassium bromate application caused a dose-dependent decrease in carrot shoot dry weight. Shtangeeva et al.^29^ determined a decrease in the biomass of wheat (*Triticum aestivum* L.), rye (*Secale cereale* L.), oat (*Avena sativa* L.) and pea (*Pisum sativum* L.) plants exposed to 50 mg/L dose of potassium bromide. Öztürk et al.^5^ determined that potassium bromate exposure caused a dose-dependent decrease in germination percentage, weight gain and root elongation in *A. cepa* bulbs.

The regressions in physiological parameters induced by potassium bromate may be associated with decreased water and nutrient uptake by plant roots. Shtangeeva et al.^29^ reported that bromine accumulation in wheat, rye and pea plant roots led to suppression of the concentrations of essential nutrients such as K, Na, Ca, Mg, Zn and Cl. This scientific data also supports our thinking. Another reason for the decrease in root elongation, which is one of the physiological parameters, is thought to be that potassium bromate reduces the mitotic cell division of *A. cepa* root meristem cells. Because the MI value decreased by 3.87% in Group IV exposed to potassium bromate at a dose of 100 mg/L compared to the control group, which also supports our opinion. In addition, there is some information in the literature that the decrease in cell division directly affects the overall plant height^30^.

### Cytogenetic effects

**Figure 3:**
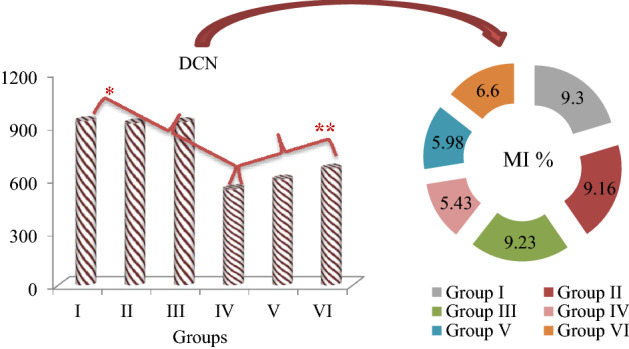
GSE and KBrO_3_ effects on dividing cell number (DCN) and MI in *A. cepa* root tip cells. Group I: Control, Group II: 465 mg/L GSE, Group III: 930 mg/L GSE, Group IV: 100 mg/L KBrO_3_, Group V: 100 mg/L KBrO_3_ + 465 mg/L GSE, Group VI: 100 mg/L KBrO_3_ + 930 mg/L GSE. *indicates the statistical difference between Groups I and IV, **indicates a statistical difference between Groups IV and VI (p < 0.05). MI rate was calculated by analyzing 10.000 cells in each group.

**Figure 4:**
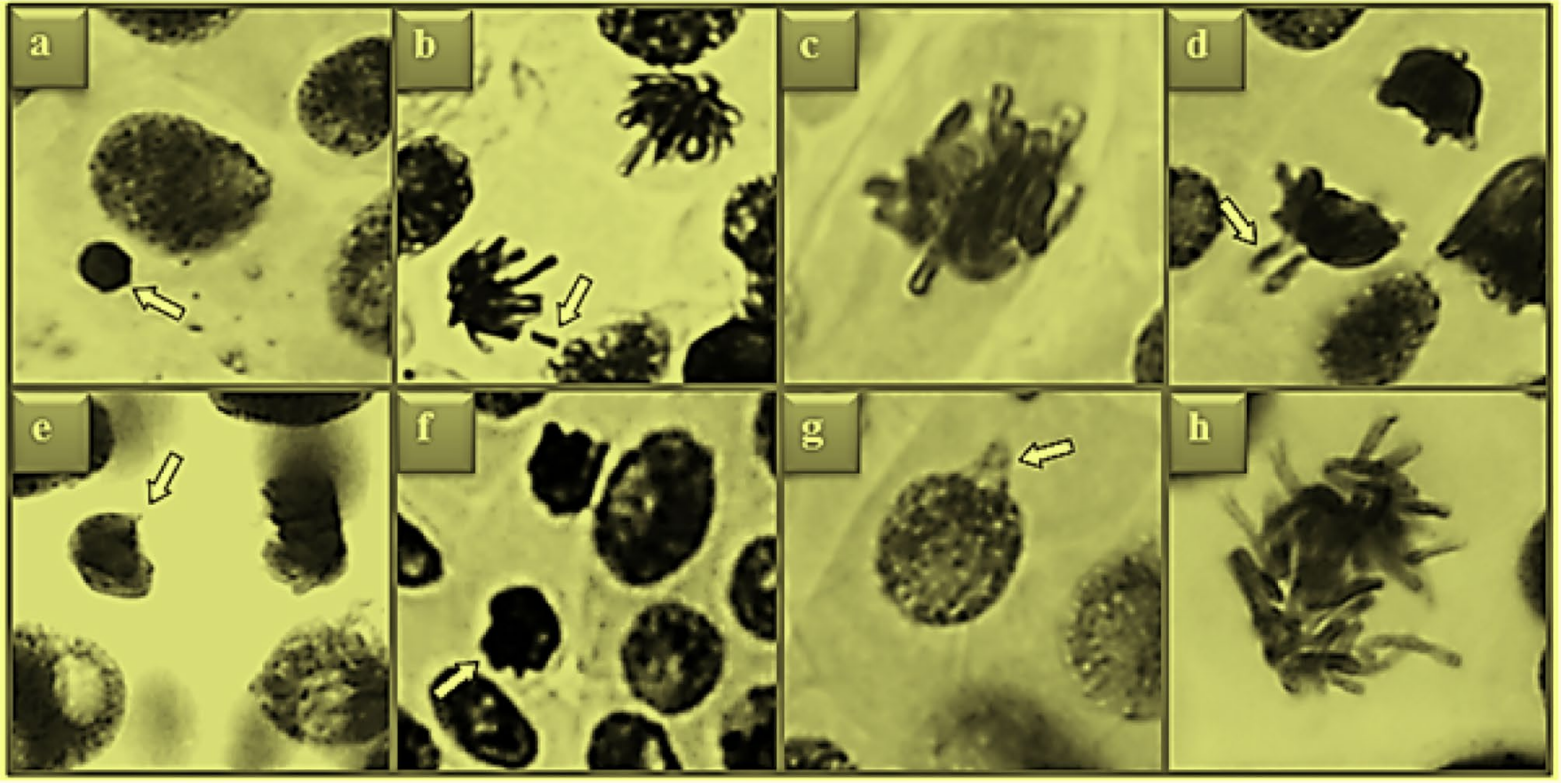
Chromosomal aberrations induced by potassium bromate. MN (**a**), fragment (**b**), sticky chromosome (**c**), vagrant chromosome (**d**), unequal distribution of chromatin (**e**), reverse polarization (**f**), nuclear bud (**g**), irregular mitosis (**h**).

The effects of potassium bromate and GSE applications on selected cytogenetic parameters are shown in Table 2, Figs. 3 and 4. The highest MI value of 9.30% was observed in the control group (Group I). It was determined that the values obtained in Group II (9.16%) and Group III (9.23%) treated with two different doses of GSE were close to the control group and there was no statistically significant difference (p > 0.05) between them. In addition, the lowest frequencies of MN and CAs were also counted in these three groups (Groups I, II and III). Potassium bromate exposure at a dose of 100 mg/L caused a statistically significant (p < 0.05) decrease in MI, and a statistically significant (p < 0.05) increase in the frequencies of MN and CAs in Group IV. Potassium bromate application induced CAs formations such as fragment, sticky chromosome, vagrant chromosome, unequal distribution of chromatin, reverse polarization, nuclear bud and irregular mitosis in root tip meristem cells. The greatest effect of potassium bromate on chromosomes occurred in the form of fragment formation with an average of 35.9. In Group V and Group VI, where potassium bromate and GSE were applied together, an increase in MI (p < 0.05) and a decrease in MN and CAs frequencies were detected (p < 0.05). These improvements observed in cytogenetic parameters were more pronounced at the dose of 930 mg/L GSE. Compared to Group IV, MI increased by 1.17%, the frequency of MN was decreased by 1.38 times and fragment frequency, which is the most observed CAs, was decreased by 1.43 times in Group VI.

**Table 2:** Protective role of GSE against KBrO_3_-induced genotoxicity.

Abnormalities	Group I	Group II	Group III	Group IV	Group V	Group VI
MN	0.17 ± 0.41^d^	0.10 ± 0.32^d^	ND^d^	61.7 ± 5.36^a^	53.4 ± 4.85^b^	44.6 ± 4.12^c^
FRG	ND^d^	ND^d^	ND^d^	51.3 ± 4.74^a^	43.8 ± 4.10^b^	35.9 ± 3.88^c^
SC	0.30 ± 0.48^d^	0.20 ± 0.42^d^	0.16 ± 0.39^d^	43.5 ± 3.98^a^	35.7 ± 3.85^b^	28.6 ± 3.42^c^
VC	ND^d^	ND^d^	ND^d^	40.6 ± 3.95^a^	33.5 ± 3.66^b^	25.4 ± 3.18^c^
UDC	0.20 ± 0.42^d^	0.10 ± 0.32^d^	0.17 ± 0.39^d^	23.8 ± 2.94^a^	15.3 ± 1.67^b^	10.2 ± 1.33^c^
RP	ND^d^	ND^d^	ND^d^	18.0 ± 1.72^a^	12.4 ± 1.35^b^	8.10 ± 1.18^c^
NB	ND^d^	ND^d^	ND^d^	13.5 ± 1.36^a^	8.60 ± 1.22^b^	4.80 ± 0.64^c^
DM	ND^d^	ND^d^	ND^d^	8.30 ± 1.20^a^	5.20 ± 0.68^b^	2.90 ± 0.42^c^

Group I: Control, Group II: 465 mg/L GSE, Group III: 930 mg/L GSE, Group IV: 100 mg/L KBrO_3_, Group V: 100 mg/L KBrO_3_ + 465 mg/L GSE, Group VI: 100 mg/L KBrO_3_ + 930 mg/L GSE. Values are shown as mean ± SD (n = 10). The MN and CAs numbers were calculated by analyzing 1.000 cells in each group. The averages shown with different letters^(a-d)^ in the same line are significant at p < 0.05. MI: mitotic index, MN: micronucleus, FRG: fragment, SC: sticky chromosome, VC: vagrant chromosome, UDC: unequal distribution of chromatin, RP: reverse polarization, NB: nuclear bud, DM: disordered mitosis, ND: no data.

The number of studies investigating the genotoxicity induced by potassium bromate in plant test materials is extremely limited. In one of these studies, Öztürk et al.^5^ found that potassium bromate application caused a dose-dependent decrease in MI and an increase in the frequency of MN in *A. cepa* root tip cells. The genotoxic effects of potassium bromate have been studied mostly in animal organisms such as humans and mice. For example, Kaya and Topaktaş^31^ reported an increase in sister chromatid exchange (SCE), CAs and MN frequency and a decrease in MI in human peripheral blood lymphocytes exposed to potassium bromate doses of 400–550 µg/mL. Al-Anazi et al.^32^ determined that the administration of potassium bromate at a dose of 150 mg/kg b.w caused an increase in the frequency of MN in peripheral blood cells and the number of liver apoptotic cells by promoting oxidative DNA damage in mice.

The increase in MN and CAs frequencies observed in root tip meristem cells of *A. cepa* exposed to potassium bromate may be associated with oxidative stress induced by potassium bromate. Especially as a result of oxidative stress induced by bromate, DNA modifications such as 7,8-dihydro-8-oxo-guanine (8-oxoG) occur. Bromate in the structure of potassium bromate does not directly react with DNA, but bromine radicals (Br*) or oxides (BrO*, BrO_2_*) cause DNA damage. Bromate induces the specific formation of 8-oxodG in the presence of -SH compounds such as glutathione (GSH) and cysteine (Cys). GSH/Cys converts potassium bromate to BrO_2_, which removes an electron from guanine. Single-electron oxidation of guanine results in the formation of 8-oxodG. Such modifications lead to DNA damage, strand breaks, and MN formations. So the mechanism of bromate-induced oxidative DNA damage is different from common types of oxidative stress such as OH*^33,34^.

### DNA-KBrO_3_ interaction confirmed by spectral shift

**Figure 5:**
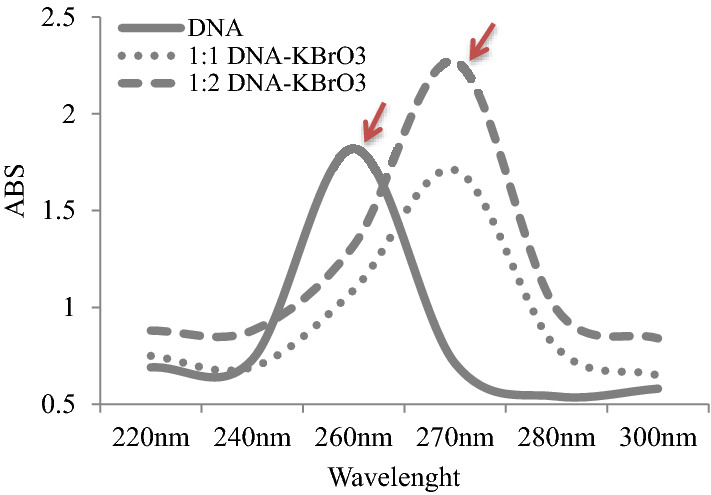
Absorption spectra of DNA in the presence of KBrO_3_ at different concentrations. Arrow shows the absorbance and wavelength changes.

CAs and MN formations induced by potassium bromate can be associated with DNA-KBrO_3_ interactions. To confirm this interaction, the spectroscopic analysis of DNA and DNA-KBrO_3_ mixture were investigated and the results are given in Fig. 5. Since the conjugated double bonds in the purine and pyrimidine rings in the DNA structure had a specific absorption peak at 260 nm, the maximum absorbance of the DNA occurred at 260 nm wavelengths. The interaction of DNA with different chemicals causes changes in the absorbance and wavelengths of the UV spectrum. In the presence of potassium bromate, there were shifts in absorbance and wavelength of DNA spectrum. The wavelength at which the maximum absorbance was obtained increased from 260 to 270 nm, and the absorbance increased from 1.82 to 2.27. An increase in wavelength indicates a bathochromic shift and an increase in absorption indicates a hyperchromic shift. Hyperchromicity seen in the UV spectrum is associated with denaturation of DNA. An increase in DNA absorbance indicates relaxation of the helix, splitting into a single-stranded form, and greater UV absorption^35^. DNA degradation and disruption of its integrity can cause MN and CAs formation and cell cycle arrests. The high frequency of MN and CAs formations as a result of potassium bromate application and the decrease in MI rates indicate cytotoxic and genotoxic effects. These effects can be explained by the dissolution of the DNA structure in the presence of potassium bromate supported by spectral shift. It has been reported in the literature that spectral shifts are observed in the DNA spectrum in the presence of various agents and these changes are associated with the deterioration of DNA integrity^36,37^.

### Biochemical effects

**Figure 6:**
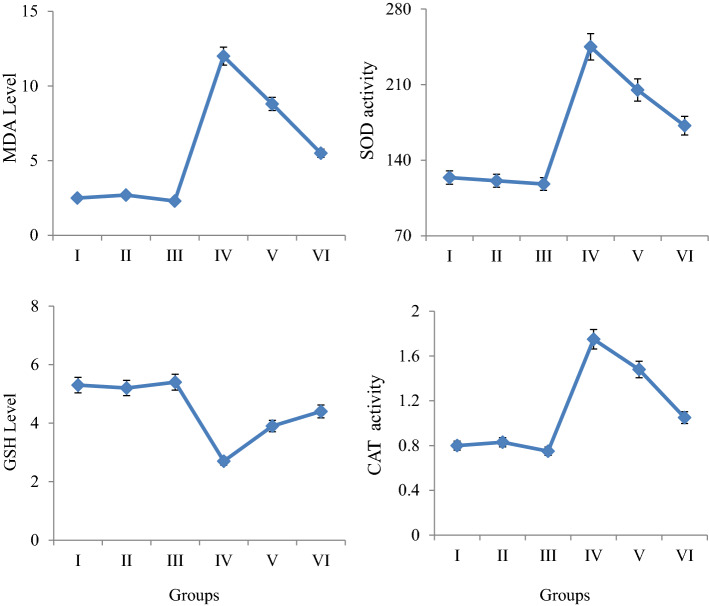
Protective role of GSE against biochemical toxicity induced by KBrO_3_. Group I: Control, Group II: 465 mg/L GSE, Group III: 930 mg/L GSE, Group IV: 100 mg/L KBrO_3_, Group V: 100 mg/L KBrO_3_ + 465 mg/L GSE, Group VI: 100 mg/L KBrO_3_ + 930 mg/L GSE. Values are shown as mean ± SD (n = 10).

The effects of potassium bromate and GSE applications on selected biochemical parameters are shown in Fig. 6. GSH, MDA, CAT and SOD levels, which are biochemical parameters, were measured, and similar levels were obtained in the control group and only GSE applied groups (p > 0.05). This result shows that GSE application alone did not cause any change in the tested parameters. Potassium bromate exposure at a dose of 100 mg/L caused statistically significant (p < 0.05) increases in MDA levels, SOD and CAT enzyme activities. Compared to the control group (Group I), MDA level increased 4.80 times, SOD activity 1.98 times and CAT activity 2.19 times in Group IV. While an increase was observed in these three parameters tested, a decrease was observed in GSH levels. GSH level decreased by 49.1% in the 100 mg/L potassium bromate-treated group compared to control. Improvements were observed in selected biochemical parameter values in Group V and Group VI, where potassium bromate and GSE were applied together. It was determined that these improvements were related to the applied GSE dose and were more pronounced in Group VI where 930 mg/L GSE was applied. In Group VI, MDA level was decreased 2.18 times, SOD activity 1.42 times, CAT activity 1.67 times decreased, GSH level increased 1.62 times compared to potassium bromate-treated group.

The number of studies investigating the biochemical toxicity induced by potassium bromate in plant test materials is extremely limited in the literature. In one of these studies, Sahin et al.^28^ found an increase in MDA level, CAT and ascorbate peroxidase (APX) enzyme activities in *Daucus carota* L. (carrot) which was applied potassium bromate at doses of 0, 0.125, 0.25, 0.50 and 1.00 mmol/kg. In addition, they did not observe a significant change in SOD activity. In another study, Öztürk et al.^5^ reported that potassium bromate application caused an increase in *A. cepa* root MDA levels, an increase in SOD and CAT enzyme activities. Studies investigating the biochemical changes promoted by potassium bromate have mostly focused on animal organisms such as rats. For example, Khan et al.^38^ found a decrease in kidney CAT, SOD, glutathione peroxidase (GPx), glutathione-S-transferase (GSTs), glutathione reductase (GR) and reduced glutathione contents in rats exposed to 20 mg/kg b.w potassium bromate. Nwonuma et al.^39^ determined an increase in MDA level, SOD and alkaline phosphatase (ALP) enzyme activities in kidney, liver and serum of rats exposed to potassium bromate at a dose of 10 mg/kg b.w orally.

MDA is a highly reactive organic compound produced during the peroxidation of unsaturated fatty acids in cell membranes or the synthesis of prostaglandin. MDA detection is used as a primary indicator of lipid peroxidation and oxidative stress. MDA reacts with amino groups of proteins and causes cross-linking in proteins. In addition, MDA can form attachments with DNA and RNA^40^. In this study, it is thought that the increase in root MDA levels as a result of exposure to potassium bromate may be due to the fact that potassium bromate damages the membranes of root meristem cells and increases the destruction of lipids in the membrane structure^28^.

Endogenous antioxidants provide a strong defense against damage such as increased oxidative stress and lipid peroxidation in cells. SOD and CAT are powerful antioxidant enzymes that protect cells against free radicals. While SOD converts superoxide radical to H_2_O_2_ and molecular oxygen, CAT converts H_2_O_2_ to water and oxygen. As a result, two toxic agents, namely the superoxide radicals and hydrogen peroxide, are converted into a harmless product^41^. In this study, it is thought that the measured increases in the SOD and CAT enzyme activities of *A. cepa* root cells exposed to potassium bromate may be due to the fact that potassium bromate promotes free radical production and increases the SOD and CAT enzyme synthesis of the cells in order to neutralize these free radicals. Because there are some studies in the literature that potassium bromate promotes oxidative stress through the production of reactive oxygen species (ROS) in different organisms and causes changes in the levels of cellular antioxidant defense system enzymes^42^. GSH, which has an important role in the endogenous antioxidant defense system of the cell, provides important protection against oxidative stress. Increasing MDA, SOD, CAT levels after potassium bromate application indicates that oxidative stress is induced while decreasing GSH level indicates that GSH is oxidized. This result shows that potassium bromate application induces oxidative stress formation and lipid peroxidation, causing deterioration in antioxidant/oxidant balance.

### Anatomical alterations

**Figure 7:**
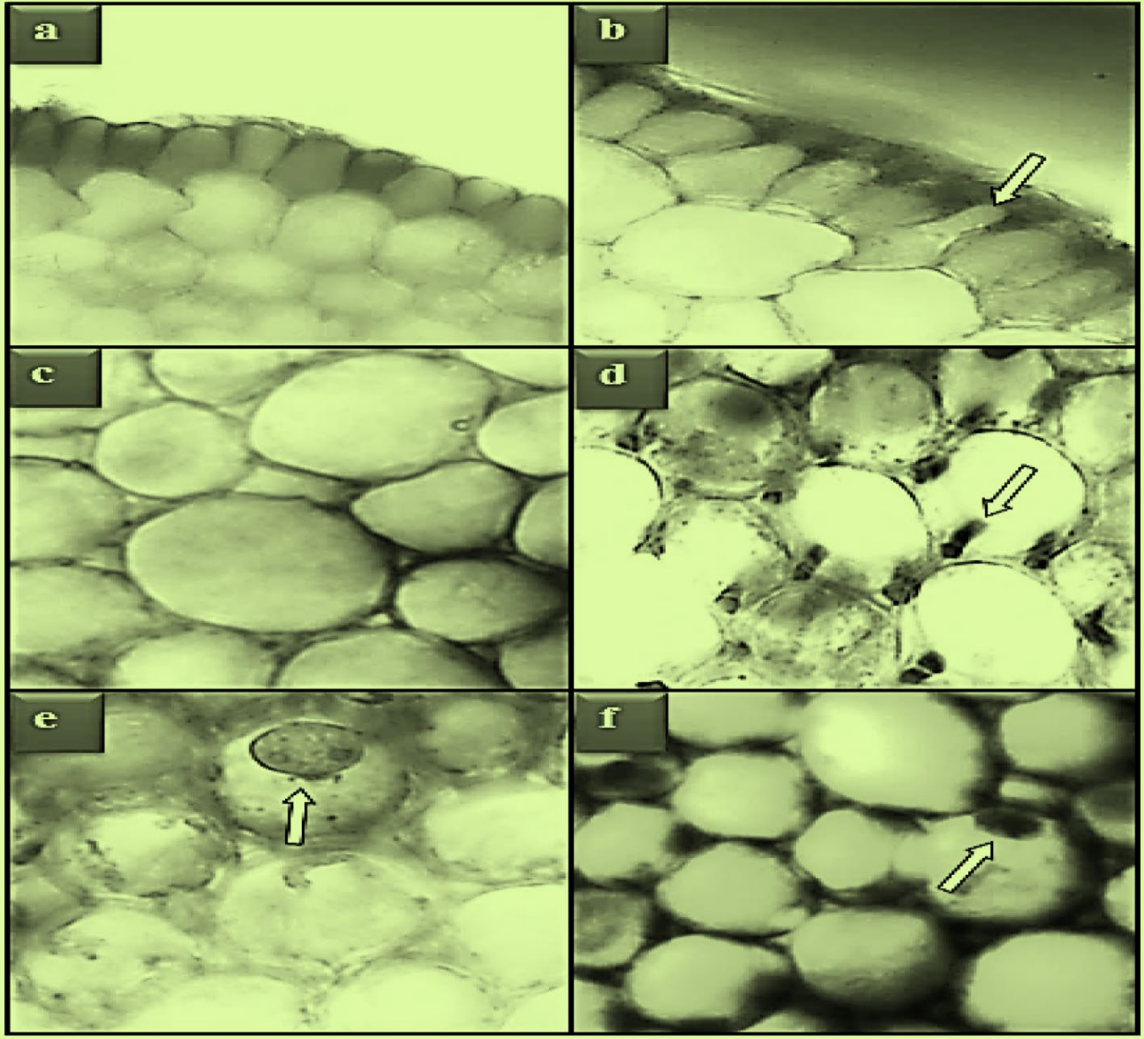
Meristematic cell damage induced by KBrO_3_. Normal appearance of epidermis cells (**a**), epidermis cell damage (**b**), normal appearance of cortex cells (**c**), thickening of the cortex cell wall (**d**), normal appearance of the cell nucleus-*oval* (**e**), flattened cell nucleus (**f**).

The anatomical damages induced by potassium bromate in root meristem cells are shown in Table 3 and Fig. 7. No anatomical damages were observed in the root tip meristem cells of control and GSE-treated groups. Anatomical damages such as epidermis cell damage, thickening of the cortex cell wall and flattened cell nucleus were observed in the root tip meristem cells of Group IV exposed to 100 mg/L dose of potassium bromate. In Group V and Group VI, where potassium bromate and GSE were applied together, a significant decrease was determined in the severity of the anatomical damages observed. It was observed that these decreases were more pronounced at 930 mg/L dose of GSE. In Group VI administered GSE at a dose of 930 mg/L, the severity of epidermis cell damage and flattened cell nucleus damage was considerably reduced. In addition, cortex cell wall thickening, which was observed as little damage (+ +) in the potassium bromate group, was not observed in Group VI.

**Table 3:** Protective role of GSE against meristematic cell damage induced by KBrO_3_.

Groups	ECD	TCCW	FCN
Group I	**−**	**−**	**−**
Group II	**−**	**−**	**−**
Group III	**−**	**−**	**−**
Group IV	**+++**	**++**	**+++**
Group V	**++**	**+**	**++**
Group VI	**+**	**−**	**+**

Group I: Control, Group II: 465 mg/L GSE, Group III: 930 mg/L GSE, Group IV: 100 mg/L KBrO_3_, Group V: 100 mg/L KBrO_3_ + 465 mg/L GSE, Group VI: 100 mg/L KBrO_3_ + 930 mg/L GSE.ECD: epidermis cell damage, TCCW: thickening of the cortex cell wall, FCN: flattened cell nucleus. (−): no damage, (+): little damage, (++): moderate damage, (+++): severe damage.

The number of studies investigating the anatomical changes induced by potassium bromate in plant test materials is extremely limited in the literature. In one of these studies, Öztürk et al.^5^ reported that potassium bromate administration caused dose-dependent anatomical damages such as necrosis, cell deformation, thickening of the cortex cell wall, accumulation of some substances in the cortex cells and flattened cell nucleus in *A. cepa* root meristem cells. There are some studies in the literature investigating the anatomical changes induced in *A. cepa* root meristem cells by some other food additives and chemical agents, although not with potassium bromate. For example, Yalçın et al.^43^ reported that tartrazine exposure at a dose of 200 mg/L induced damage such as unclear vascular tissue, epidermis cell damage, flattened cell nucleus and cortex cell wall thickening in *A. cepa* root tip meristem cells. Kurt et al.^44^ determined that the application of 1,4 dioxane at a dose of 100 mg/L caused anatomical changes such as epidermis cell deformation, thickening of the cortex cell wall and flattened cell nucleus in *A. cepa* root meristem cells.

Anatomical damages caused by exposure to potassium bromate can be explained by some physical defense mechanisms developed to prevent potassium bromate from entering the cell. Because, in the observations made under the research microscope, a significant increase in the number of epidermis cells of the roots exposed to potassium bromate was detected. This defense mechanism developed by the roots to prevent the entry of potassium bromate into the cell may have increased the contact of the cells with each other and ultimately caused a mechanical press. This mechanical pressure can cause deformations in the epidermis cells and the nuclei of these cells. The information in the literature that plants develop physical defense mechanisms based on phytochemical (such as terpenoids, alkaloids, polypeptides, phenolic compounds, phytoalexins, phytoanticips) and anatomical (increase in the number and order of epidermis/cortex cells, thickening of the cell wall) changes based on the synthesis and accumulation of some natural compounds to protect them from the negative effects of different toxic agents supports this idea^45–47^.

### Recovery effects of GSE

**Figure 8:**
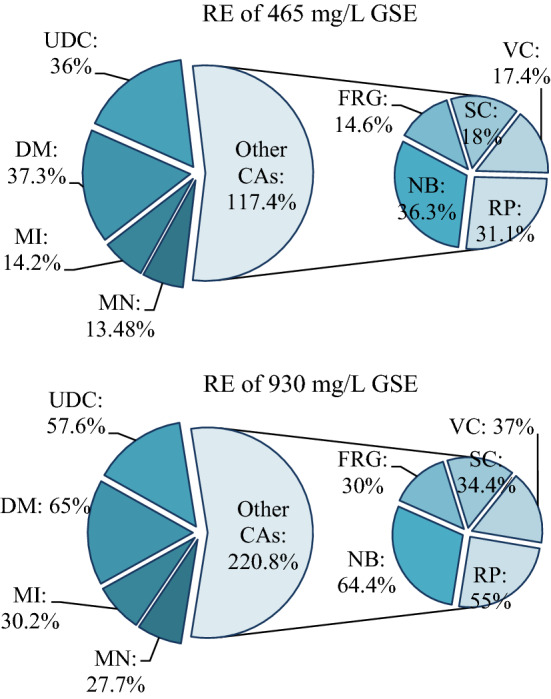
RE of 465 mg/L and 930 mg/L GSE against genotoxic effects of KBrO_3._ MI: mitotic index, MN: micronucleus, FRG: fragment, SC: sticky chromosome, VC: vagrant chromosome, UDC: unequal distribution of chromatin, RP: reverse polarization, NB: nuclear bud, DM: disordered mitosis.

In recent years, various plant extracts such as nettle, turmeric, ginger, bitter melon, sage, green tea and green coffee have been used in studies to reduce the toxicity caused by chemical agents. In this study, two different doses of GSE (465 mg/L and 930 mg/L) were used to reduce the multiple toxicities induced by potassium bromate in *A. cepa*. GSE extract reduced the toxicity induced by potassium bromate and caused improvement in the investigated physiological, cytogenetic, biochemical and anatomical parameters. It was determined that this improvement was directly related to the applied GSE dose (Fig. 8). 465 mg/L GSE treatment in Group V resulted in 14.2% improvement in MI, 13.48% decrease in MN frequency, and 14.6%-36% reduction in CAs frequencies. Administration of 930 mg/L GSE in Group VI provided 30.2% improvement in MI, 27.2% reduction in MN frequency, and 30–65% decrease in CAs frequency.

The improvement observed in all parameters examined as a result of GSE application can be explained by the antioxidant nature of GSE. Components such as proanthocyanin, catechin, epicatechin, linoleic acid, oleic acid, linolenic acid, palmitoleic acid, fetocopherol, tocotrienol, flavan-3-ol, phenolic acid, anthocyanin, flavonol, hydroxycinnamic acid, β-sitosterol and sterol in the content of GSE protects the cells against internal and external free oxygen radicals by providing strong antioxidant properties^11,12^. It is known that potassium bromate induces oxidative stress, causing cytotoxicity, genotoxicity, mutagenicity and biochemical toxicity. In this study, the deterioration in antioxidant/oxidant balance in the potassium bromate administered group confirms this claim. GSE reduced the toxic effects induced by potassium bromate with its antioxidant role in reducing oxidative stress.

There are some studies in the literature demonstrating this protective role of GSE in *A. cepa*. For example, Kalefetoğlu Macar et al.^48^ reported that GSE had a therapeutic effect against cobalt (Co) toxicity in *A. cepa*. In a similar study, Kalefetoğlu Macar et al.^15^ determined that 150 and 300 mg/L doses of GSE had a protective role against cobalt (II) nitrate stress in *A. cepa* root tip cells. In another study, Yılmaz et al.^16^ observed that GSE has a reducing role in benzyl benzoate genotoxicity in *A. cepa.*

## Conclusion

In this study, it was determined that high-dose potassium bromate caused multiple toxicity in *A. cepa*. Potassium bromate induced oxidative stress, causing regressions in physiological development, cytogenetic abnormalities and anatomical changes. In addition, it was determined that GSE provided improvement by reducing the toxicity caused by potassium bromate, and it was determined that this improvement increased depending on the dose. In conclusion, *A. cepa* test material is a reliable biological indicator for toxicity determination. Potassium bromate, which is widely used as an additive in many sectors today, induces multi-faceted toxicity in a eukaryotic living system, and therefore its use should be restricted or used at as low levels as possible. An alternative solution to these toxicities is the application of herbal extracts with antioxidant effect. GSE exhibited a protective role by reducing the toxicity of potassium bromate and it was determined that it could be a toxicity-reducing solution. This study draws attention to the fact that chemical agents contaminating the environment adversely affect organisms and that natural herbal extracts can be a solution against toxicity.

## Data Availability

The datasets used and/or analyzed during the current study are available from the corresponding author on reasonable request.
